# Colour-Blind Sailors and the Board of Trade

**Published:** 1896-11-14

**Authors:** 


The Hospital

A Journal of

The Medical Sciences and Hospital Administration, with

AN EXTRA NURSING SUPPLEMENT.

Vol. XXI.—No. 529.] [Nov. 14, 1896.

### Colour-blind Sailors and the Board of Trade.

The case of colour-blind sailors imperatively needs to be stated to a justice-loving public. It has been dealt with by a Royal Society Commission at the instigation of the Board of Trade, and it has also been dealt with by the British Medical Association. But, up to the present time, colour-blind sailors are as great a danger to the sea-going world as they always were, and the Board of Trade is as callous and cruel in its treatment of a meritorious, but unfortunate, class of seamen as it always has been.

What is the case of colour-blind sailors, from their own point of view, and from the point of view of modern medical science? A very instructive, and also a very distressing, answer is given to this question in a small pamphlet just published by Mr. T. H. Bickerton, Ophthalmic Surgeon to the Liverpool Royal Infirmary, and a consistent champion of the otherwise almost friendless colour-blind sailor. Good sight is one of the most indispensable of all a sailor's endowments. Defective sight, more especially a sight defective in its appreciation of colours, absolutely unfits a man for the important and essential duty of keeping a safe "look-out" for his ship during the night. It is the fact that a very large proportion of all the collisions which take place at sea are due to the defective eyesight of those particular seamen who happen at the moment to be keeping the look-out. But, insists the man of mere plain sense, there ought to be no such thing as a colour-blind sailor. Surely medical science can tell us whether a boy be colour-blind or not before he is sent to sea; and it would be the simplest thing in the world to have every intending sailor boy examined before he decides upon a seafaring career; and, therefore, the simplest thing in the world to put an end to the dangers arising from colour-blindness in sailors, and that without injury to anybody.

Most assuredly this judgment of common sense is literally and exactly correct; but yet, and notwithstanding the absolute ease of prevention, hundreds of lives are periodically lost at sea in consequence of defective eyesight in sailors, and large numbers of young and middle-aged sailors are constantly being deprived of occupation and subsistence for no fault, but only because of a defective eyesight, which, if they had been examined in youth, would have effectually prevented them from ever being sailors at all. What and where, then, is the exact difficulty in consequence of which all who "go down to the sea in ships" are still exposed to such tremendous risks every night whilst they may be compelled to remain at sea? This is the exact difficulty—that the Board of Trade refuses to insist upon the examination of the would-be sailor-boy by a competent scientific specialist before he is allowed to be apprenticed to the sailor's calling.

But the danger to the public is not all. The seamen have to be considered, and it is difficult to speak without indignation of the injustice to which they are subjected under the present rules. It will hardly be believed, but it is nevertheless the fact, that, indispensable as is perfect sight to every seafaring man, no compulsory examination is ever made of the eyes until a young sailor goes up for his second mate's certificate. By that time, of course, he has been several years at sea, is an adult man, and is absolutely committed to the sailor's calling. He goes up for his second mate's certificate at this period of his life, and then, for the first time, the colour-blind tests are presented to him, and if he fails in the due distinguishing of colours, he is at once *ipso facto* discharged from his calling and thrown helpless, and in many cases penniless and friendless, upon the world. Not only so, but the persons who make the examinations in eyesight are, as a rule, absolutely without competence to do so, any more than the first man who may be met in the street. Not only are they not "eye specialists," they are not even doctors.

If details of the hardships and sufferings resulting from these regulations are asked for, the reader is referred to Mr. Bickerton's pamphlet.

Now let the just reader consider these terrible evils, and then, on the other hand, recollect how easily they might be prevented altogether, and with absolute certainty. The one and only thing that is necessary is to make regulations which will render the examination of the eyesight by a competent specialist compulsory before the proposing sailor is allowed to take the first step in his intended career. As a matter of fact the very first practical step should be this compulsory examination by a competent specialist of the boy's eyesight. One would think—every right-minded person cannot but think—that to merely state a case of this kind is to ensure the prompt carrying out by the Board of Trade of what is so obviously necessary and right. Not at all. The Board of Trade, approached again and again by all classes of persons and in all manner of ways, still insists upon doing absolutely nothing at all except what it has previously done. Then the Board of Trade must take the responsibility. True; but what of the ruined fortunes and broken hearts of our British sailors? Ought an indignant public to tolerate this wicked and cruel state of things any longer? Let the general press take the matter up as it has done in the past, and let the public answer for itself.

